# Recent advances in molecular pathology of craniopharyngioma

**DOI:** 10.12688/f1000research.11549.1

**Published:** 2017-07-24

**Authors:** Sarah Larkin, Niki Karavitaki

**Affiliations:** 1 Nuffield Department of Clinical Neurosciences, University of Oxford, Department of Neuropathology, John Radcliffe Hospital, Oxford, OX3 9DU, UK; 2 Centre for Endocrinology, Diabetes, and Metabolism, Birmingham Health Partners, Birmingham, B15 2TH, UK; 3 Institute of Metabolism and Systems Research, College of Medical and Dental Sciences, University of Birmingham, Birmingham, B15 2TT, UK

**Keywords:** Craniopharyngioma, pharmacological treatment, pituitary, molecular pathology, BRAF mutations

## Abstract

Craniopharyngiomas are rare epithelial tumours arising along the path of the craniopharyngeal duct. Two major histological subtypes have been recognised, the papillary and the adamantinomatous. Craniopharyngiomas remain challenging tumours to manage and are associated with significant morbidities and mortality. Recent advances in the molecular pathology of these neoplasms have identified *BRAF *mutations in the papillary variant, offering promising options for targeted pharmacological treatment. The involvement of β-catenin and the Wnt pathway in the tumorigenesis of the adamantinomatous subtype has been previously established with the identification of stabilising mutations in exon 3 of *CTNNB1*. Further understanding of the pathogenesis of this subtype has been facilitated with the use of mouse models and xenograft experiments. It has been proposed that the clusters of cells with upregulated Wnt/β-catenin signalling induce tumour formation in a paracrine manner; the complex interactions occurring between different cell populations need to be further clarified for further expansion of this hypothesis. This review outlines recent key advances in our understanding of the molecular pathology of craniopharyngiomas and discusses some of the challenges that need to be overcome for the development of targeted therapies that will hopefully improve the management and the outcomes of these patients.

## Introduction

Craniopharyngiomas (CPs) are epithelial tumours (WHO grade I) arising along the path of the craniopharyngeal duct (embryonal structure connecting stomodeal ectoderm and the evaginated Rathke’s pouch). Recent epidemiological data suggest incidence rates of 0.17–0.2 cases per 100,000 people ^1– 4^. They show a bimodal age distribution with peak incidence rates in children aged 5–14 and adults aged 50–74 ^5^. No gender differences have been reported ^3, 4^.

Histologically, two primary subtypes have been recognised, the adamantinomatous (aCP) and the papillary (pCP). The aCP is the most common and macroscopically shows cystic and/or solid components, necrotic debris, fibrous tissue, and calcification (especially common in children). The margins of aCP are sharp and irregular, often making the identification of the surgical planes difficult. The cytoarchitecture of aCP comprises a palisaded basal layer of small cells; above this, there is an intermediate layer of variable thickness composed of loose aggregates of stellate cells (termed “stellate reticulum”) and a top layer facing into the cyst lumen with abruptly enlarged, flattened, and keratinised flat plate-like squamous cells. The flat squames are desquamated singly or in distinctive stacked clusters, forming nodules of wet keratin, often heavily calcified and apparent grossly as white flecks ^5, 6^. The pCP has almost exclusively been described in adults (14–50% of adult cases and only up to 2% in children). Macroscopically, it tends to be solid or mixed with cystic and solid components, calcification is rare, and the cyst content is usually viscous and yellow. It is generally well circumscribed, and infiltration of adjacent brain tissue by neoplastic epithelium is less frequent than in aCP or even absent. Microscopically, it is composed of mature squamous epithelium forming pseudopapillae and of an anastomosing fibrovascular stroma without the presence of peripheral palisading of cells or stellate reticulum ^5, 6^.

The optimal management of CPs remains challenging, and the main options include surgery combined or not with radiotherapy; nonetheless, they can show aggressive and unpredictable behaviour with recurrence(s) difficult to treat ^7^. Furthermore, these tumours are associated with significant long-term morbidity (mainly involving endocrine, visual, hypothalamic, neurobehavioural, and cognitive sequelae) and mortality, attributed to the damage of critical structures by the primary or recurrent tumour and/or to the adverse effects of therapeutic interventions ^8– 11^.

Better understanding of the molecular pathology of CPs is of major importance for the development of targeted therapies aiming to improve the outcomes of these patients. In recent years, there have been significant advances in this field, and we summarise them in this brief review.

## Mutations in *BRAF* are characteristic for pCP, while aCP is associated with *CTNNB1* mutations

The involvement of β-catenin and the Wnt pathway in the tumorigenesis of aCP has been established, since stabilising mutations in exon 3 of *CTNNB1* that prevent phosphorylation and degradation of β-catenin have been identified in aCPs ^12^. However, until recently, the genetic event underlying the development of pCP had remained unclear. Several studies employing whole exome sequencing, next-generation panel sequencing, pyrosequencing, and Sanger sequencing have demonstrated the presence of activating mutations in *BRAF* (V600E) in pCPs. Their reported prevalence varies according to the sequencing method used and ranges from 81 to 100% ^13– 16^. Extensive analysis of aCPs using the same methods has not identified recurrent mutations other than those in exon 3 of *CTNNB1* ^13– 15^. The lack of genetic complexity seen in these tumours is typical of their benign behaviour, and the almost-perfect segregation of mutation with tumour variant ^16^ coupled with the existence of a *BRAF* V600E mutation-specific antibody (VE1) provides a useful diagnostic tool for specimens with scant epithelium or where diagnosis is challenging; it should be noted that there is some concern about the specificity of this antibody in pituitary tissue, necessitating cautious interpretation ^17– 19^. Much more promising, however, is the possibility for targeted pharmacological treatment with agents directed against the *BRAF* (V600E) mutation.

## *BRAF* (V600E) mutations offer the possibility for targeted pharmacological treatment

The finding that most pCPs harbour a *BRAF* (V600E) mutation has opened up the exciting possibility of repurposing existing drugs for the treatment of pCP cases refractory to surgery and radiotherapy. Pharmacological agents that specifically target and inhibit mutant *BRAF* (V600E) are very effective in malignant tumours positive for this mutation. Although the development of resistance to these agents is a potential drawback, the addition of other inhibitors of the MAPK pathway (e.g. trametinib, which inhibits MEK) can increase their efficacy ^20^ and reduce the risk of cutaneous squamous-cell carcinoma, a common complication of BRAF inhibitor treatment.

Two recent reports have shown significant reduction in tumour volume in cases of treatment-refractory pCP. Aylwin and colleagues ^21^ described a patient with progressive visual deterioration due to a recurrent pCP previously managed with three surgeries and fractionated radiotherapy. The tumour harboured *BRAF* (V600E) mutation and the patient was treated with vemurafenib. Stabilisation of vision was achieved within two weeks, along with a dramatic reduction in tumour size. Near-complete radiological remission occurred after three months, but the patient developed cerebrospinal fluid leak, pneumocephalus, and meningitis, necessitating antimicrobial therapy and surgical repair. Treatment was interrupted after three months, but the CP recurred within six weeks and vemurafenib was restarted. Tumour growth was stabilised until seven months after treatment initiation when progressive regrowth was detected. The second report from Brastianos and colleagues ^22^ described a patient with multiply recurrent pCP with *BRAF* (V600E mutation). Four surgical decompressions were unsuccessful at controlling the growth of the tumour, which had a large cystic component. The patient suffered bilateral optic neuropathy and panhypopituitarism. Treatment was initially dabrafenib monotherapy, and after 17 days the solid and cystic components decreased by 50% and 70%, respectively. At day 21, the MEK inhibitor trametinib was added for a further 14 days to reduce the likelihood of resistance to BRAF inhibition. This regime led to solid and cystic components of the tumour decreasing in total by 85% and 81%, respectively. Subsequently, the patient underwent endoscopic transsphenoidal resection followed by radiotherapy and has remained symptom free 18 months after radiation.

Both cases illustrate the promising potential for the use of BRAF (V600E) inhibitors and remain to be validated with clinical trials.

## aCP and pCP have different epigenomic and transcriptional signatures

As well as their genetic differences, CP variants have been shown to have different epigenomic and transcriptomic signatures. Analysis of the most variably methylated CpG sites by both unsupervised hierarchical clustering and principal component analysis identified two distinct methylation clusters that separated aCP and pCP ^15^. Additionally, unsupervised consensus clustering using the most variably expressed genes identified by microarray analysis resulted in clear separation of the two subtypes, indicating that they likely have distinct gene expression signatures ^15, 23^. Analysis of mRNA expression demonstrated up-regulation of Wnt/β-catenin pathway targets in aCP ( *LEF1* and *AXIN2*) and also components of the hedgehog signalling pathway ( *GLI2*, *PTCH1*, and *SHH*). Additionally, the stem cell marker *PROM1* (encoding CD133) was overexpressed. These findings are in keeping with previous published data from the same group that suggest the activation of Wnt and sonic hedgehog (SHH) pathways in the cell clusters of aCP (see below) ^24– 28^. For a detailed discussion of the opportunities for therapy targeting these upregulated pathways, see 29.

Interestingly, neither the methylation nor the mRNA expression signatures of paediatric aCP and adult aCP separated when analysed as described above, suggesting that they may not be distinct at the epigenomic or transcriptional level ^15^.

## Cluster cells have a functional role in the promotion of invasion

The cells that accumulate β-catenin in aCP are small in number and often, but not always, accumulate to form small clusters with a whorl-like pattern near the infiltrating edges of the tumour ^26, 30– 35^. The function of these cluster cells remains enigmatic, but they represent a population strikingly different from the bulk of the tumour.

A mouse model of aCP demonstrated that *CTNNB1* mutation in Rathke’s pouch progenitor cells is sufficient to drive aCP tumorigenesis. Expression of *CTNNB1* lacking exon 3 (degradation-resistant β-catenin) under the control of Hesx1 in the early stages of mouse pituitary development results in the formation of tumours that closely resemble human aCP, although they lack some of the regressive changes seen in the human tumour, e.g. wet keratin. Mutation of β-catenin in undifferentiated Rathke’s pouch progenitors is sufficient to drive tumorigenesis of aCP, but once lineage commitment has occurred, the mutation is no longer tumorigenic ^36^. Interestingly, although *CTNNB1* mutation was present in the whole population of progenitor cells, there was only cytosolic and/or nuclear accumulation of β-catenin in the characteristic cell clusters near the invading tumour edge ^36^, indicating that an event other than *CTNNB1* mutation determined which cells formed clusters.

Hölsken *et al.* harvested cluster cells from human aCP using laser capture microdissection and sought to determine whether they harboured an activating mutation in exon 3 of the β-catenin gene and whether the Wnt pathway was activated by quantifying *Axin2* and *BMP4.* The cluster cells expressed mutated *CTNNB1*, along with the surrounding tumour cells, but only the cluster cells expressed elevated levels of Axin2 and BMP4 RNA and protein. The authors concluded that *CTNNB1* mutation is not sufficient to drive nuclear β-catenin accumulation ^26^. These cluster cells were also found to express *Fascin*, a Wnt target gene with a role in cytoskeletal organisation and cell migration ^24, 26^. Downregulation of both β-catenin and fascin expression in human primary aCP tumour cells using siRNA impaired their motility and migrational capacity ^24^. The study did not separate cells derived from different features of the aCP prior to culture, so the culture was likely to contain non-cluster cells as well as the cluster cells the authors sought to characterise. Mutation in *CTNNB1* was present in 66% of aCPs, but its effect on the behaviour of the cells was not explored ^24^. However, the observation that siRNA-mediated downregulation of β-catenin or fascin impairs motility in primary human aCP cells suggests a potential role for β-catenin, fascin, and (by inference) the cluster cells in the invasion into surrounding brain. Additionally, the same group demonstrated that claudin, a tight-junction component, was expressed in the finger-like protrusions of aCP but was absent from the cell clusters; this pattern of expression is consistent with a migratory phenotype for the cluster cells. Furthermore, inhibition of claudin expression increased significantly the motility of aCP tumour cells, and claudin expression was significantly lower in invasive than in non-invasive aCPs ^27^.

Further insight into the role of cluster cells in the pathogenesis of aCP was provided in a study by Stache *et al.*, who used a xenotransplant model of human aCP in immunodeficient mice. Serial sectioning of a whole aCP xenograft provided a reconstruction of the formation of the finger-like protrusions that invade surrounding brain. From these serial sections, the authors inferred that the cluster cells themselves extrude through the palisading epithelial layer that encases the tumour to infiltrate the surrounding brain and then become enclosed again by a palisading epithelial layer to form the whorls characteristic of the finger-like protrusions of aCP. Consistent with this model for infiltration, the xenografts and human tumour specimens showed increased proliferation of the palisading cells and expression of p21 ^WAF/Cip1^ in the cluster cells, indicating cell cycle arrest. Although this model is a snapshot of the developing aCP and not a time course, the mechanism proposed is an attractive explanation for both the motile and the quiescent phenotype of cluster cells as well as their sustained presence in the infiltrating tumour projections.

## Pituitary stem cells have a paracrine role in the pathogenesis of aCP

The cluster cells in aCP are a clearly distinct subpopulation, but their function within the tumour is not yet well understood. There is growing evidence for a population of anterior pituitary stem cells expressing Sox2 that can give rise to the main anterior pituitary progenitor cell lineages and that, crucially, also have tumour-inducing potential ^30, 36^. Andoniadou *et al.* created a mouse model that expresses degradation-resistant β-catenin in Sox2 ^+^ cells upon tamoxifen induction to determine whether these cells could give rise to tumours at the embryonic stage and also in adult mice. The embryonic-induced mice developed tumours similar to those seen in the Hesx1 model ^36^, with identifiable β-catenin-accumulating cluster cells reminiscent of those in human aCP that were quiescent or slow-dividing (did not express Ki-67) and were undifferentiated (did not express lineage commitment markers αGSU and PIT1) ^37^. In the adult-induced model, the pattern of β-catenin expression was more complex, with some cells showing nucleocytoplasmic accumulation.

Lineage tracing experiments used Sox2 ^+^ cells simultaneously expressing both degradation-resistant β-catenin and yellow fluorescent protein (YFP) aiming to identify both these tumour progenitor cells and their descendants ^37^. Interestingly, after induction of degradation-resistant β-catenin, the majority of tumour cells formed did not express YFP. However, there were populations of YFP-expressing cells adjacent to the tumour cells, some of which formed clusters. These findings indicate that the tumour cells were not derived from Sox2 ^+^ cells expressing degradation-resistant β-catenin. However, nucleocytoplasmic accumulation of β-catenin was observed in tumour cells that did not express YFP (and so were not derived from the Sox2 ^+^ progenitor cells), suggesting that this accumulation and activation of the Wnt pathway is non-cell-autonomous and may occur as a result of signalling from the adjacent Sox2 ^+^ cell population. Indeed, the β-catenin-accumulating, YFP ^–^ tumour cells were often found in close proximity to the YFP ^+^ cells, suggesting a functional relationship ^37^.

Expression of degradation-resistant β-catenin in Sox2 ^+^ cells leads to the formation of β-catenin-accumulating cell clusters that resemble those in human aCP, implicating Sox2 ^+^ progenitor cells as the cells in which the tumour-initiating mutation in β-catenin occurs. However, these cells do not give rise in an autonomous manner to the tumour cells as would be expected if they were true mutation-sustaining cancer stem cells. Instead, they appear to drive tumour formation in a paracrine manner by inducing tumorigenic events in adjacent cells. The authors propose that induction of degradation-resistant β-catenin initiates a brief period of proliferation in a proportion of Sox2 ^+^ cells. The resulting daughter cells then form the β-catenin-accumulating clusters that are characteristic of aCP. These clusters become quiescent and secretory, signalling by means of secretory proteins including members of the fibroblast growth factor (FGF), transforming growth factor beta (TGFβ), epithelial growth factor, and SHH pathways, along with pro-inflammatory cytokines and chemokines, ^30^ to induce transformation in surrounding cells and perhaps modify the tumour microenvironment. It is these non-Sox2 ^+^-derived cells that form the bulk of the tumour ^37^.

**Figure 1.**
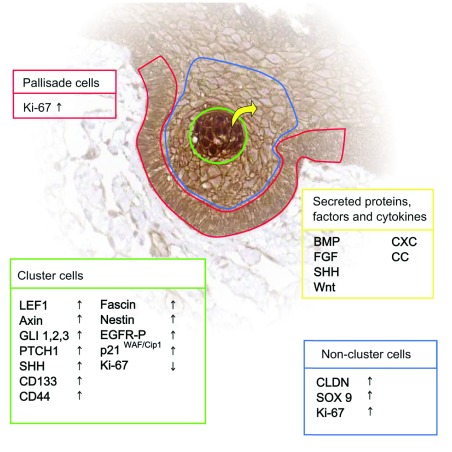
Subpopulations of cells at the invading edge of adamantinomatous craniopharyngioma. The relative expression of various targets and activity of signalling pathways vary according to cell type. BMP, bone morphogenetic protein; FGF, fibroblast growth factor; SHH, sonic hedgehog.

The mouse paracrine model of aCP pathogenesis includes a stem-cell-like progenitor that sustains the oncogenic mutation but is not the cell-of-origin of the tumour (comprehensively reviewed in 38– 40). There is considerable evidence to suggest that human aCP may follow the same pathogenic route. The cluster cells of human aCP are non-proliferative and express members of the FGF, TGFβ, and SHH pathways ^25, 26, 30^, and they are also non-proliferative and undifferentiated ^36, 41^. A representation of the different cell populations at the invading edge of aCP and some of the differentially expressed proteins and factors is shown in Figure 1. The tumour environment is a competitive one in which more proliferative cells will out-compete and replace their more indolent neighbours in a process termed somatic or clonal evolution (extensively reviewed in 42). This concept generally refers to aggressive tumours with a high degree of genetic inhomogeneity, in which particular mutations may confer survival advantages to subpopulations of cells, but it seems reasonable that, even in a tumour with a limited mutational landscape, a quiescent population of cells would quickly be replaced by proliferating cells unless they were performing a function essential for the pathogenesis of the tumour, as has been proposed by other authors ^38, 40^. Instead, even in highly invasive and destructive aCPs that destroy surrounding hypothalamic and pituitary tissue, clusters of β-catenin-accumulating, quiescent cells remain. Furthermore, these clusters are often located near to the invading tumour periphery, in regions where the proliferation of surrounding cells is high and selective pressure is likely to be significant. Reports differ as to the prevalence of *CTNNB1* mutation in aCPs and sequencing of a bulk aCP tumour is likely to result in false negative results if the mutation is present only in cluster cells, which may constitute a small proportion of the bulk tumour and so fall below the limit of detection of the method employed. However, immunohistochemical studies have suggested that even in aCP with no obvious cell clusters, there are β-catenin-accumulating cells found distributed throughout the tumour ^35^. This is useful from a diagnostic perspective but may also be indicative of the importance of these cells for tumour pathogenesis. Further experiments will be required to determine whether cluster cells are essential paracrine signalling hubs for tumour pathogenesis after the tumour-initiating mutation in *CTNNB1*, but the current available evidence is consistent with this model.

## Mouse models, primary cell culture, and xenografts offer opportunities to study aCP pathogenesis

Mouse models, primary cell cultures, and xenograft models are valuable tools for studying the pathogenesis of aCP, each of which has advantages for answering particular questions; however, none fully recapitulates the features of the human tumour. The mouse models (both Hesx1- and Sox2-driven ^36, 37^), while they allow the study and manipulation of tumorigenesis, lack some features of human aCP: for instance, they do not develop a defined palisading layer of epithelial cells at the invading edge of the tumour, nor do they show calcification or the anucleated ghost cells (wet keratin) that are pathognomonic for human aCP. It has been suggested that wet keratin is a feature of a more mature tumour and that the lack of this feature in the mouse models is likely a consequence of the relatively short period (weeks) over which the mouse tumour develops. A report by Scagliotti *et al.* ^43^ of an intrauterine-diagnosed congenital aCP at 23 weeks of gestation described the presence of wet keratin and calcium deposits in the tumour. This is comparable to the 2–5-month period over which the Sox2 ^+^ aCP mouse model develops ^37^, so perhaps the lack of wet keratin and calcification in the mouse tumour is due to species-specific differences in the pathogenesis of this tumour or perhaps the mouse model is lacking a facet of the human tumour that is not yet identified.

Primary human aCP cell cultures ^24^ have the advantage of being derived from the human tumour and can be used to screen pharmacological agents relatively easily; however, the process of tissue disruption required for cell culture means that the complex architecture of the aCP is lost. Any functional relationship between the cluster cells and surrounding tumour is likely to be perturbed or destroyed. Furthermore, culture conditions cannot replicate microenvironmental signals to the tumour from surrounding tissue. It has not been tested whether aCP primary cells in culture develop cluster-like formations and the cultures have not been molecularly profiled. Greater characterisation of this model is needed to determine the extent of its utility in studies of human aCP.

Xenograft models ^28^ preserve the cytoarchitecture of the human aCP and provide a microenvironmental context (albeit from a different species) in which the aCP can grow and invade. However, opportunities for the manipulation of tumour pathogenesis are limited in this model, and its suitability for testing pharmacological agents may be compromised by the low availability of human tumour material and differences in responses to agents between species.

All the models discussed here have provided greater insight into the pathogenesis of aCP and represent useful tools for answering outstanding questions, but the particular advantages and disadvantages of each must be acknowledged and considered.

## Heterogeneity of aCP represents a challenge to the development of pharmacological treatment

This review has highlighted the very different architecture and pathogenesis of CP variants. While the discovery of the *BRAF* (V600E) mutation in pCP has afforded us an opportunity for a promising pharmacological intervention, recent progress in understanding aCP reveals a morphologically complex tumour with distinct cell populations that have differing functions and cells of origin. If the paracrine model for aCP tumorigenesis withstands further investigation, it presents a potential target for therapeutic intervention, namely the cluster cells that initiate and support tumorigenesis in surrounding cells. However, the stem-like properties of these cells make them difficult targets for cytotoxic agents, which are often ineffective against quiescent cells. Any targeted therapy aimed at disrupting the paracrine signalling between clusters and surrounding cells will require deeper understanding of the signalling events and the effect on the surrounding tumour.

## Conclusions

Understanding of CP pathogenesis has increased considerably in recent years. The identification of *BRAF* (V600E) mutations in pCP has led to the potential for new targeted pharmacological therapy. Our understanding of aCP has been greatly improved by mouse model and xenograft experiments revealing new and critical roles for β-catenin-accumulating cluster cells; however, there is still much to clarify about the complex interactions that occur between different cell populations within this neoplasm before we are in the position to develop effective targeted therapies.
